# Effects of Rearing Conditions, Geographical Origin, and Selection on Larval Diapause in the Indianmeal Moth, *Plodia interpunctella*

**DOI:** 10.1673/031.012.11901

**Published:** 2012-10-17

**Authors:** Leanage K. W. Wijayaratne, Paul G. Fields

**Affiliations:** ^1^ Department of Entomology, University of Manitoba, 12 Dafoe Road, Winnipeg, Manitoba, Canada R3T 2N2; ^2^ Cereal Research Centre, Agriculture and Agri-Food Canada, 195 Dafoe Road, Winnipeg, Manitoba, Canada R3T 2M9

**Keywords:** photoperiod, temperature

## Abstract

The Indianmeal moth, *Plodia interpunctella* (Hübner) (Lepidoptera: Pyralidae), is a serious insect pest of stored products, and its late-instar larvae diapause as pre-pupae. Diapause induction in *P. interpunctella* was investigated for four populations obtained from Modesto, California, U.S.A.; Vancouver, British Columbia, Canada; and two locations from Winnipeg, Manitoba, Canada. Insects were reared at 25° C and 16:8 L:D for 9 days. The larvae were then either continuously maintained under those conditions or transferred to 25° C 8:16 L:D, 20° C 16:8 L:D, or 20° C 8:16 L:D, and the percent diapause was recorded. In the experiment with four populations, the highest diapause frequency was observed at 20° C 8:16 L:D. The two Winnipeg populations had significantly higher frequency of diapause than the California populations, indicating the increased frequency of diapause in populations from higher latitudes.

In a second experiment, the Vancouver population was selected for diapause. Larvae were reared at 25° C 16:8 L:D for 9 days, then placed at 20° C 8:16 L:D for the rest of their development, and percent diapause was determined. Eggs laid by moths that completed diapause in this first (parental) generation were used to obtain a second generation (F1), and the experiment was repeated as in the first generation. Selection increased the frequency of diapause to 91%, compared to 26% in the unselected population, after selecting over two generations. The narrow sense heritability of selection in *P. interpunctella* was 0.39 in the first selection, and 0.82 in the second. This study has shown that both low temperature and short photoperiod are required to induce diapause in North American populations of *P. interpunctella*, and that selection can increase diapause in a few generations.

## Introduction

Indianmeal moth, *Plodia interpunctella* (Hübner) (Lepidoptera: Pyralidae), is an insect pest of stored products, and is found throughout the world (Rees 2004). It causes infestations in a wide array of commodities (Johnson et al. 1992; Johnson et al. 1995; Sedlacek et al. 1996; Nansen and Phillips 2003; Nansen and Phillips 2004; Nansen et al. 2004). This species undergoes larval diapause (Bell and Walker 1973) as a pre-pupa (Prevett 1971). Diapause induction in *P. interpunctella* depends on a number of factors: photoperiod (Bell and Walker 1973; Kikukawa et al. 2005), temperature (Tsuji 1958; Tzanakakis 1959; Prevett 1971; Bell 1976a; Mbata 1987), age of larvae exposed to diapause-inducing conditions (Bell 1976a), origin of population (Bell 1976a), larval density (Tsuji 1959a; Tsuji 1960), and type of food (Williams 1964).

Previous studies explored the effects of ecological factors on diapause induction in *P. interpunctella* (Tsuji 1958, 1959a, 1959b; Bell 1976a; Mbata 1987). Some of these studies examined the effects of temperature and photoperiod on diapause induction in this species (Bell et al. 1979; Mbata 1987). Generally, *P. interpunctella* from temperate regions have greater frequency of diapause than those from tropical regions (Bell et al. 1979). However, there is little information on populations from northern areas of North America, where high frequency of diapause would be predicted according to Bell et al. (1979). There are several examples of diapause responding to selection (Tauber et al. 1986; Danks 1987), but very little information is available on such effects in *P. interpunctella*. Diapause in *P. interpunctella* increased or decreased with selection (Tsuji 1960). However, the effects of this selection under various temperatures and photoperiods was not examined The objectives of our experiment were to study the effect of temperature and photoperiod on diapause induction in four populations of *P. interpunctella* from geographically different regions of North America, and to examine the effect of selection on frequency of diapause under various rearing conditions that include both temperature and photoperiod.

## Materials and Methods

### Diapause induction

Larvae of *P. interpunctella* populations from Modesto, California, U.S.A. (37° 38′ 21″ N, 120° 59′ 44″ W); Vancouver, British Columbia, Canada (49° 15′ 0″ N, 123° 8′ 0″ W); and two locations from Winnipeg, Manitoba, Canada (49° 53′ 0″ N, 97° 10′ 0″ W) were used in this experiment. Insects were reared at 25° C, 16:8 L:D (using fluorescent lights), and 40–50% relative humidity, on a rearing medium prepared by mixing 1000 g cracked wheat, 40 g brewer's yeast (ICN Biomedicals, Inc., Aurora, OH, U.S.A.), 50 g wheat germ, 2 g sorbic acid (Sigma-Aldrich, Inc., http://www.sigmaaldrich.com/), 2 g methyl-p-hydroxybenzoate (Sigma-Aldrich, Inc.), 120 mL honey, 120 mL of glycerol, and 60 mL of water. To collect eggs, fifty adults comprised of both sexes were placed in an empty 900 mL glass bottle, covered with a nylon mesh, and placed upside down on a plastic Petri dish (Bell 1976a). Eggs were inspected under a microscope, and only plump eggs were used. Eggs collected within a 24 hour period were placed, in batches of 50 eggs, in 900 mL glass bottles with 200 g of rearing medium. Cultures were maintained for a few generations before the start of the experiments.

The developing insects were maintained at 25° C and 16:8 L:D for 14 days before the bottles were placed in one of four conditions: 25° C with 16:8 L:D, 25° C with 8:16 L:D, 20° C with 16:8 L:D, or 20° C with 8:16 L:D. Eggs took 4–6 days to hatch at 25° C 16:8 L:D; therefore, larvae were approximately nine days old at the time of transfer. There were 2– 4 bottles, or 100 to 200 individuals, for a given treatment. Larvae developed under the above conditions, and adults emerged. Seven days following the end of the adult emergence under each condition, the rearing media were dissected, and the presence of pre-pupae, dead larvae, dead pupae, and dead adults were recorded. Live pre-pupae were considered to be in diapause.

### Diapause selection

Bottles with 50 eggs from the Vancouver population were held in 200 g of the medium at 25° C 16:8 L:D for 14 days before they were transferred to 20° C 8:16 L:D, and held until emergence of non-diapausing adults was assumed to be completed. The emerged adults were counted and removed every 2–3 days. Ten days after the last adult emerged, the bottles were placed at 2.5° C in total darkness, and held for 6 weeks to terminate diapause of pre-pupae in the rearing medium (Bell 1976b). This insured that adults would emerge all at the same time and allow mating. Following this 6-week cold exposure, bottles were returned to 25° C and 16:8 L:D, and adults that subsequently emerged were counted and considered to have undergone diapause. These adults were held in empty glass jars, and their eggs (F_1_) were collected and tested for diapause induction as described for the parental generation. Eggs from the adults that completed diapause in the F_1_ (second) generation were used to start an F_2_ (third) generation. Frequency of diapause in this F_2_ generation was compared with that in unselected progeny derived from the original (Vancouver) population that was maintained continuously at 25° C 16:8 L:D. Frequency of diapause in selected and unselected groups was investigated using the methods previously described involving exposure to four sets of conditions: 25° C 16:8 L:D, 25° C 8:16 L:D, 20° C 16:8 L:D, or 20° C 8:16 L:D. As above, seven days after adult emergence ceased in each rearing jar, the rearing mediums were dissected in order to count the remaining insects.

### Data analysis

The frequency of insects in diapause in each replicate was transformed using the square root of arcsine transformation. These transformed data were used in ANOVA (SAS Institute 2002–2008). In the diapause induction experiment, the effects of treatment (combination of temperature and photoperiod) and population (Vancouver, California, Winnipeg 1, Winnipeg 2) on diapause induction were analyzed. For a given population, the means of the frequency of diapausing insects under different treatments were compared using Tukey's multiple range test, or Student's t-test (*p* = 0.05). In the selection experiment, the effects of selection on diapause frequency in the F1 and F_2_ generations were analyzed using ANOVA. An ANOVA was also performed to compare final diapause frequency in the selected population to final diapause frequency in the unselected population. This ANOVA was followed by Tukey's multiple range tests (*p* = 0.05). Narrow sense heritability was calculated by employing the diapause selection data in the following equation: Hn^2^ = P/S, where Hn^2^ = heritability in the narrow sense, R = response to selection, and S =selection differential (Russell 2009).

**Table 1.**
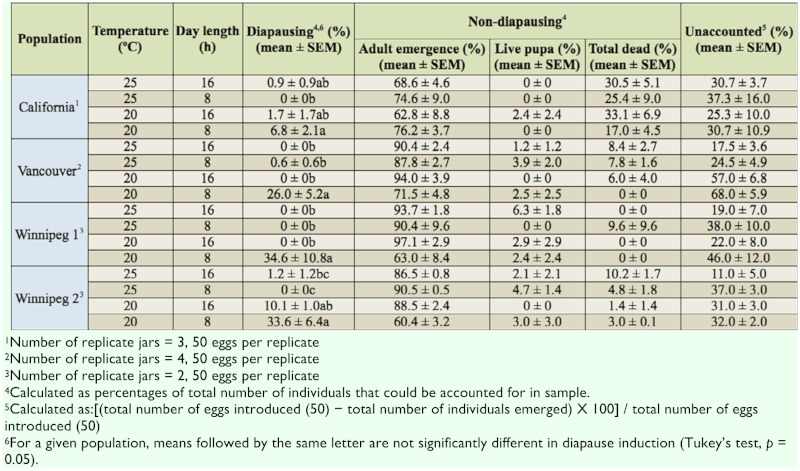
Percent diapausing and non-diapausing *Plodia interpunctella* from different geographical populations.

## Results

### Diapause induction

The frequency in diapause significantly differed among treatments (ANOVA, F_3;28_ = 88.3, *p* < 0.0001) and populations (ANOVA, F_3_,_28_ = 6.54, *p* = 0.0017). The interaction of treatment by population was also significant (ANOVA, F_9,28_ = 5.18, *p* = 0.0004). In all the populations, maximum diapause frequency was observed at 20° C 8:16 L:D (Table 1). In the Vancouver and Winnipeg 1 populations, diapause frequency at 20° C 8:16 L:D differed significantly from that in all other conditions. In the Winnipeg 2 and California populations, frequency of diapause was highest at 20° C 8:16 L:D, but was not significantly different from all other treatments.

There were no significant differences in diapause frequency by decreasing only the photoperiod from 16 to 8 h at 25° C (Table 1). Similarly, decreasing only the temperature from 25° C to 20° C without decreasing the photoperiod from 16 h did not significantly change the diapause frequency. However, when both the temperature and photoperiod were reduced (from 25° C 16:8 L:D to 20° C 8:16 L:D), there was a significant increase in diapause frequency in all four populations.

When only data from 20° C 8:16 L:D were analyzed, significant differences in the frequency of diapause were evident among populations that originated in different geographical locations (ANOVA, F_3,7_ = 6.20, *p* = 0.0220). Diapause frequency in Winnipeg 1 and Winnipeg 2 was significantly different from the California population (Tukey's, *p* = 0.05). However, diapause frequency in Vancouver population was not significantly different from any of the other three populations (Tukey's, *p* = 0.05).

Between 11% and 68% of the insects were unaccounted (did not mature to late-instar larvae). This could be due to infertile eggs, or mortality in the early instars that would not be detected during dissections of rearing mediums.

**Table 2.**
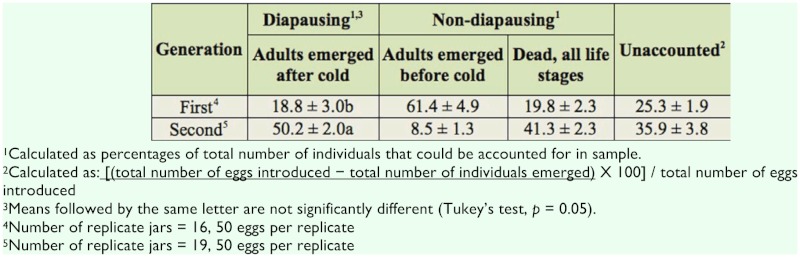
Percent diapausing and non-diapausing *Plodia interpunctella* (Vancouver population) reared at 25° C with 16:8 L:D for 14 days, transferred to 20° C with 8:16 L:D, exposed to 2.5° C for 6 weeks, and selected over two generations.

**Table 3.**
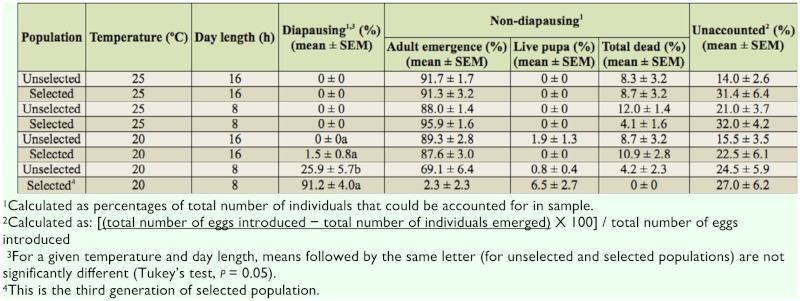
Final comparison for diapause induction between selected progeny of second generation (Table 2) of Vancouver population with its unselected original population (n = 4).

### Diapause selection

There was a significant change in percentage of adults emerging after cold treatment from the F_1_ to the F_2_ generation (ANOVA, F_1,33_ = 58.82, *p* < 0.0001; Table 2). When this selected population was tested with the unselected original population, there was only a significant diapause induction at 20° C 8:16 L:D. Under these conditions, the selected population gave the highest percent diapause (91.2%), while the unselected population gave 25.9% (ANOVA, F_1,6_ = 44.78, *p* = 0.0005; Table 3). There were no differences in the adult emergence between the selected and unselected populations when tested at 20° C 16:8 L:D (ANOVA, F_1,6_ = 2.98, *p* = 0.1350), 25° C 16:8 L:D, and 25° C 8:16 L:D. ANOVA could not be conducted in the last two treatment conditions because the diapause did not change from zero. These results showed that selection increased diapause in *P. interpunctella* over two generations.

Narrow sense heritability (Hn^2^) (Russell 2009) was estimated at 0.39 for the first generation, and 0.82 for the second generation. For the first generation, the selection differential was 81.2 (selected parents diapause = 100%, unselected parents diapause = 18.8%; Table 2), the response to selection was 31.4 (progeny from selected parents diapause = 50.2%, unselected parents diapause = 18.8%; Table 2), resulting in a heritability of 0.39 (response to selection/selection differential). Similar calculations were done for the second generation.

## Discussion

In the experiment with four populations, diapause induction mostly took place at 20° C and 8:16 L:D. This result was also seen in the selected population, which had over 90% diapause in the same conditions, but no diapause at the other conditions. Bell et al. (1979) showed that 6 out of 23 populations had at least 20% diapause when two-day-old larvae were transferred from 25° C with 15:9 L:D to 20° C with 15:9 L:D. Also, the diapause frequency increased to 47% when the photoperiod was reduced to 11:13 L:D. These results are similar to our findings that the maximum diapause induction took place when late-instar larvae were transferred from 25° C with 16:8 L:D to 20° C with 8:16 L:D, although the diapause frequency varied from 7 to 34% among four populations. Bell et al. (1979) found that there was no diapause induction at 25° C 15:9 L:D, and some diapause in a few populations at 25° C 9:15 L:D. Similarly, in the four populations we tested, diapause frequency was negligible both at 25° C 16:8 L:D and 25° C 8:16 L:D. Mbata (1987) showed that the transfer of *P. interpunctella* fourth-instar larvae from 30° C to 20° C gave different diapause frequencies based on the photoperiod to which they were exposed. They found 0% diapause under continuous light, and 37% under complete darkness.

In our study, in a given population, reducing only the photoperiod from 16 to 8 h at 25° C did not increase diapause frequency. Also, in a given population, the diapause frequency was not changed by changing only the temperature (transferring larvae from 25° C 16:8 L:D to 20° C 16:8 L:D). In contrast, three out of four populations (Vancouver, Winnipeg 1 and Winnipeg 2) increased diapause frequency when both the temperature and photoperiod were decreased. Although not significantly different, the fourth population (California) also had a trend to increase diapause frequency due to a similar change in temperature and photoperiod. This shows that the decrease in either photoperiod or temperature alone does not trigger diapause frequency in *P. interpunctella*, but requires the concurrent change in both factors. There have been some studies that used similar conditions to induce diapause in *P. interpunctella*, as tested in the current study. *Plodia interpunctella* larvae reared at 25° C, 16:8 L:D were induced to diapause when transferred to 20° C 8:16 L:D. The percentage that entered diapause varied with the age at which the larvae were transferred (Bell 1976a). In a later study, Bell et al. (1979) tested the effects of changing both the temperature and photoperiod independently and in combination. However, in that study, changing the temperature and photoperiod to which the larvae were exposed was done within relatively a short time following hatching (approximately within two days). In our study the larvae were transferred to low temperature and short photoperiod approximately nine days after hatching. Diapause frequency of different larval instars in response to changing environmental conditions is important as it provides information on their survival under unfavorable conditions as well.

The two populations from a higher latitude (Winnipeg 1 and Winnipeg 2) had greater diapause frequency than the population from a lower latitude (California). The population from Vancouver was not significantly higher than California, but showed similar diapause frequency as the Winnipeg populations. This is in agreement with Bell et al. (1979), which showed that populations from higher latitudes had higher frequencies of diapause than populations from lower latitudes.

Different criteria have been used to determine the diapause status in *P. interpunctella*. Mbata (1987) used the time taken from egg hatch to adult emergence as a criterion for diapause (23–34 days at 30° C, or 60–72 days at 20° C as the non-diapausing condition). Tsuji (1958) recorded fully-grown-arrested larvae as the indicator of diapause, compared to the non-diapausing larvae that pupated quickly. Bell (1976a) considered the larvae that were fully grown, but did not pupate within two weeks, to be in diapause. These differences in the criteria for determining the diapausing status may have contributed to the variation in interpretation of results.

Tsuji (1960) studied the effect of selection for diapause in *P. interpunctella* larvae, showing that *P. interpunctella* reared at 30° C had 24% diapause when first collected from the field, but this declined to 1% after 3 years of continuous rearing at 30° C (photoperiod unspecified). When larvae were reared at 20° C with 11:13 L:D, most entered diapause. By selecting non-diapause individuals over several generations, the diapause frequency dropped to 0.4% when reared at 20° C with 11:13 L:D. In contrast, selecting for diapausing individuals maintained a population, with almost all individuals entering diapause at 20° C with 11:13 L:D. Similar to Tsuji (1960), we showed that selecting for diapausing individuals over several generations caused an increase in diapause frequency. However, Tsuji (1960) did not examine the combined effects of temperature and photoperiod. The work in our study showed that both low temperature and short photoperiod were needed to induce diapause, even in selected populations.

There are several studies on the effects of selection on diapause (Henrich and Denlinger 1982; Tauber and Maski 1986; Danks 1987; Schmidt and Paaby 2008). In some cases there was no effect. At 20° C, selection for diapause in *Ephestia cautella* for three generations gave diapause frequencies between 26% and 29% (Cox et al. 1981). In contrast, selection of *Diatraea grandiosella* increased the percentage diapause from 56% to 100% over three generations (Takeda and Chippendale 1982). In *Sarcophaga argyrostoma*, crossing of a fast developing strain with a slow developing strain resulted in a population with an intermediary development time (Bradley and Saunders 1985).

The narrow sense heritability of selection in *P. interpunctella* varied between 0.39 and 0.82 in this study. *Oncopeltus fasciatus* has a heritability of 0.70 (Dingle et al. 1977; Danks 1987) and *Heliothis zea* a heritability of 0.77 for time required to emerge following diapause (diapause intensity) (Holtzer et al. 1976; Danks 1987). In *Drosophila melanogaster*, selection for ten generations from a mass-bred population maintained in the lab for upward direction resulted in the heritability of 0.12 (Gilchrist and Huey 1999). Our study shows that the heritability for diapause selection in *P. interpunctella* is not fixed, but can widely vary between generations.

This study has shown that both low temperature and short photoperiod, simulation of autumn conditions, are required to induce diapause in North American populations of *P. interpunctella*. This has implications for control, as diapause has shown to increase cold tolerance (Naeemullah et al. 1999, Fields and Timlick 2010), so insects in diapause would be harder to control with low temperature. Diapausing insects are also more resistant to insecticides (Bell 1977). This study shows that diapause frequency can be increased within a few generations at low temperatures and short photoperiods. Thus *P. interpunctella* populations from southern latitudes or heated warehouses could quickly adapt to locations with cool temperatures and short photoperiods. Therefore, renewed attention needs to be paid to diapause phenomenon in *P. interpunctella* in order to determine diapause capacity in field populations, and to test the possibility of manipulating temperature and light in storage conditions in order to prevent diapause, and perhaps render insects more susceptible to control.

## References

[bibr01] Bell CH (1976a). Factors governing the induction of diapause in *Ephestia elutella* and *Plodia interpunctella* (Lepidoptera).. *Physiological Entomology*.

[bibr02] Bell CH (1976b). Factors influencing the duration and termination of diapause in the warehouse moth, *Ephestia elutella*.. *Physiological Entomology*.

[bibr03] Bell CH (1977). Toxicity of phosphine to the diapausing stages of *Ephestia elutella, Plodia interpunctella* and other lepidoptera.. *Journal of Stored Products Research*.

[bibr04] Bell CH, Bowley CR, Cogan PM, Sharma S (1979). Diapause in twenty-three populations of *Plodia interpunctella* (Hübner) (Lep., Pyralidae) from different parts of the world.. *Ecological Entomology*.

[bibr05] Bell CH, Walker DJ (1973). Diapause induction in *Ephestia elutella* (Hübner) and *Plodia interpunctella* (Hübner) (Lepidoptera, Pyralidae) with a dawn-dusk lighting system.. *Journal of Stored Products Research*.

[bibr06] Bradley HK, Saunders DS (1985). The selection for early and late pupariation in the flesh-fly, *Sarcophaga argyrostoma*, and its effect on the incidence of pupal diapause.. *Physiological Entomology*.

[bibr07] Cox PD, Mfon M, Parkin S, Seaman JE (1981). Diapause in a Glasgow strain of the flour moth, *Ephestia kuehniella*.. *Physiological Entomology*.

[bibr08] Danks HV (1987). Variation in response.. *Insect Dormancy: an Ecological Perspective*..

[bibr09] Fields PG, Timlick B, Carvalho MO, Fields PG, Adler CS, Arthur FH, Athanassiou CG, Campbell JF, Fleurat-Lessard F, Flinn PW, Hodges RJ, Isikber AA, Navarro S, Noyes RT, Riudavets J, Sinha KK, Thorpe GR, Timlick BH, Trematerra P, White NDG (2010). The effect of diapause, cold acclimation and ice-nucleating bacteria on the coldhardiness of *Plodia interpunctella*.. *Proceedings of the 10th International Working Conference on Stored-Product Protection*.

[bibr10] Henrich VC, Denlinger DL (1982). Selection for late pupariation affects diapause incidence and duration in the flesh fly, *Sarcophaga bullata*.. *Physiological Entomology*.

[bibr11] Johnson JA, Wofford PL, Whitehand LC (1992). Effect of diet and temperature on development rates, survival and reproduction of the Indianmeal moth (Lepidoptera: Pyralidae).. *Journal of Economic Entomology*.

[bibr12] Johnson JA, Wofford PL, Gill RF (1995). Developmental thresholds and degree-day accumulations of Indianmeal moth (Lepidoptera: Pyralidae) on dried fruits and nuts.. *Journal of Economic Entomology*.

[bibr13] Kikukawa S, Rou R-R, Sugimoto M (2005). Effect of light pulses in early scotophase on resetting of the night-measuring diapause clock of the Indian meal moth, *Plodia interpunctella*.. *Physiological Entomology*.

[bibr14] Mbata GN (1987). Studies on the induction of larval diapause in a Nigerian strain of *Plodia interpunctella* (Hübner) (Lepidoptera: Pyralidae).. *Insect Science and Its Application*.

[bibr15] Naeemullah M, Tanaka K, Tsumuki H, Takeda M (1999). Relationship of cold tolerance to developmental determination in the Indianmeal moth, *Plodia interpunctella* (Lepidoptera: Phycitinae).. *Applied Entomology and Zoology*.

[bibr16] Nansen C, Phillips TW (2003). Ovipositional responses of the Indianmeal moth, *Plodia interpunctella* (Hübner) (Lepidoptera: Pyralidae) to oils.. *Annals of the Entomological Society of America*.

[bibr17] Nansen C, Phillips TW (2004). Attractancy and toxicity of an attracticide for Indianmeal moth, *Plodia interpunctella* (Lepidoptera: Pyralidae).. *Journal of Economic Entomology*.

[bibr18] Nansen C, Phillips TW, Palmer MW (2004). Analysis of the insect community in a stored-maize facility.. *Ecological Research*.

[bibr19] Prevett PF (1971). Some laboratory observations on the development of two African strains of *Plodia interpunctella* (Hübn.) (Lepidoptera: Phycitidae), with particular reference to the incidence of diapause.. *Journal of Stored Products Research*.

[bibr20] Rees D. (2004). *Insects of Stored Products*..

[bibr21] Russell PJ (2009). *iGenetics: A Molecular Approach*..

[bibr22] SAS Institute. (2002–2008). The SAS System for Windows, Release 9.1..

[bibr23] Schmidt PS, Paaby AB (2008). Reproductive diapause and life-history clines in North American populations of *Drosophila melanogaster*.. *Evolution*.

[bibr24] Sedlacek JD, Weston PA, Barney J, Subramanyam B, Hagstrum DW (1996). Lepidoptera and Psocoptera.. *Integrated Management of Insects in Stored Products*,.

[bibr25] Tauber MJ, Tauber CA, Masaki S (1986). *Seasonal Adaptations of Insects*..

[bibr26] Takeda M, Chippendale GM (1982). Environmental and genetic control of the larval diapause of the southwestern corn borer, *Diatraea grandiosella*.. *Physiological Entomology*.

[bibr27] Tsuji H (1958). Studies on the diapause of the Indian-meal moth, *Plodia interpunctella* Hübner. I. The influence of temperature on the diapause, and the type of diapause.. *Japanese Journal of Applied Entomology and Zoology*.

[bibr28] Tsuji H (1959a). Studies on the diapause of the Indian-meal moth, *Plodia interpunctella* Hübner. II. The effect of population density on the induction of diapause.. *Japanese Journal of Applied Entomology and Zoology*.

[bibr29] Tsuji H (1959b). Studies on the diapause of the Indian-meal moth, *Plodia interpunctella* Hübner. III. The influence of high temperature on the inception of diapause in the 20°C nondiapause stock.. *Japanese Journal of Applied Entomology and Zoology*.

[bibr30] Tsuji H (1960). Studies on ecological life history of the Indian-meal moth, *Plodia interpunctella*. 1. Comparative studies of the three stocks with special reference to the onset of diapause.. *Japanese Journal of Applied Entomology and Zoology*.

[bibr31] Tzanakakis ME (1959). An ecological study of the Indian-meal moth *Plodia interpunctella* (Hübner) with emphasis on diapause.. *Hilgardia*.

[bibr32] Williams GC (1964). The life-history of the Indian meal-moth, *Plodia interpunctella* (Hübner) (Lep. Phycitidae) in a warehouse in Britain and on different foods.. *Annals of Applied Biology*.

